# Musculoskeletal Symptoms and Risk of Burnout in Child Care Workers — A Cross-Sectional Study

**DOI:** 10.1371/journal.pone.0140980

**Published:** 2015-10-21

**Authors:** Peter Koch, Johanna Stranzinger, Albert Nienhaus, Agnessa Kozak

**Affiliations:** 1 Centre of Excellence for Epidemiology and Health Services Research for Healthcare Professionals (CVcare), University Medical Centre Hamburg-Eppendorf, Martinistrasse 52, 20246 Hamburg, Germany; 2 Health Protection Division (FBG), Institution for Statutory Accident Insurance and Prevention in the Health and Welfare Services (BGW), Pappelallee 33, 22089 Hamburg, Germany; CSIR-Indian Institute of Toxicology Research, INDIA

## Abstract

**Objectives:**

German child care workers' job satisfaction is influenced by the consequences of unfavourable underlying conditions. Child care workers tend to suffer from psychosocial stress, as they feel that their work is undervalued. The objective of the present study is to investigate how the psychosocial factors of the effort-reward imbalance (ERI) model influence musculoskeletal symptoms (MS) and the risk of burnout. To our knowledge this is the first study investigating the association between the factors of the ERI model and MS in child care workers.

**Methods and Findings:**

Data from 199 child care workers were examined in a cross-sectional study. Psychosocial factors were recorded with the ERI questionnaire. MS was recorded with the Nordic Questionnaire and risk of burnout with the *Personal Burnout* scale of the Copenhagen Burnout Inventory. Multivariate analysis was performed using linear and logistic regression models. The response rate was 57%. In most of the sample (65%), an effort-reward imbalance was observed. 56% of the child care workers were at risk of burnout and 58% reported MS. Factors associated with risk of burnout were *subjective noise exposure* (OR: 4.4, 95%CI: 1.55–12.29) and *overcommitment* (OR: 3.4; 95%CI: 1.46–7.75). There were statistically significant associations between MS and *overcommitment* (low back pain—OR: 2.2, 95%CI: 1.04–4.51), *low control* (overall MS OR: 3.8; 95%CI: 1.68–3.37) and *risk of burnout* (overall MS OR: 2.3, 95%CI: 1.01–5.28). For *ERI* no statistically significant associations were found with reference to risk of burnout or MS.

**Conclusion:**

Overcommitment in child care workers is related to MS and risk of burnout. There is also evidence that *low control* is associated with MS and *subjective noise exposure* with risk of burnout. *Effort-reward imbalance* is not related to either outcome. This occupational health risk assessment identifies changeable working factors in different types of facilities.

## Introduction

Burnout is a common phenomenon among employees in the service sector [1]. The occupational group of child care workers is no exception to this—either in Germany or elsewhere [2–6]. Studies in Germany have shown that 10–30% of this occupational group exhibit burnout symptoms or are at risk of burnout [7–9]. Factors associated with burnout in this group include low income, lack of recognition [10, 11] and high noise exposure [12–14]. Low work-associated resources are also associated with burnout. Studies have shown that low control is associated with higher degrees of burnout [15, 16]. The psychosocial situation of employees can be recorded using the factors of the effort-reward imbalance model (ERI model). In the ERI model [17], employee health is related to performance and reward. If there is imbalance between these factors, a stress situation arises (ERI) and the risk of stress-associated diseases increases. Recent German studies have found ERI prevalence values of between 64% and 67% in child care workers [18, 19]. In child care workers and teachers, ERI is strongly correlated with burnout [16]. Overcommitment is a personality trait in the ERI model and is also associated with the risk of burnout in several occupations [20].

International studies have found that child care workers are at increased risk of musculoskeletal symptoms (MS) [8, 21–23]. Both biomechanical and psychosocial factors play a role in the development of MS. Longitudinal studies in several occupational groups have found relevant associations between effort-reward imbalance and MS [24–26]. On the other hand, a review of the association between ERI and MS in all occupations concluded that this association was inconsistent [27]. To our knowledge there have been no studies on this association in child care workers to date.

The primary objective of the present study is to investigate the effect of ERI on MS in child care workers, after allowing for physical stress. The secondary objective is to identify the factors related to the risk of burnout in child care workers.

## Material and Methods

As part of occupational risk assessment, a funding provider for facilities for children and adolescents in Hamburg carried out continuous stress monitoring in September 2014. In this study we present the results of a cross-sectional analysis; a follow-up is planned for 2015.

The funding provider bears the responsibility for caring for children in three different types of facility:

1. Day-care centres for children, in which care is provided to children aged up to 6 years.
2. School co-operations: School children in full-time schools are cared for during the afternoon, when there is no teaching.
3. Facilities to support children and adolescents: these include sheltered housing groups and projects for adolescents.

A questionnaire was developed for monitoring and was distributed to 400 employees in 26 different facilities of the funding provider, in collaboration with the works council. Bearing in mind the subsequent follow-up, the questionnaire was performed in a pseudo-anonymous form. The study was agreed with the data safety officer of the funding provider. The Hamburg Ethics Committee specifically approved this study (reference number: PV4792). Written informed consent was given by all participants.

All employees who worked for at least ten hours per week were invited to take part in the study. In order to reach employees who were on leave at the time of data collection, or who were often absent for other reasons, the participants were allowed four weeks to complete and return the questionnaires.

Aside from demographic information, the questionnaire included information on area of work, weekly working hours, period of employment, everyday working life situations, physical stress, subjective noise exposure, psychosocial factors and work-related resources. The outcomes of risk of burnout and MS were ascertained.

*Physical stress*, such as lifting or carrying children, was ascertained with selected questions in a validated instrument [28]. A cumulative score could then be calculated from five items (values 5–20), and this records physical stress with respect to *awkward body postures*, *standing*, *sitting*, *and lifting and carrying children*. *As a predictor this score* was then coded into three different degrees, using the tertile boundaries.

*Subjective noise exposure* was assessed with 13 questions we had developed ourselves. Items such as: “This level of noise bothers me” or “There are rooms where I hear particularly poorly” were answered on a 5-point scale, ranging from *strongly agree* to *strongly disagree*. A cumulative score was calculated from the answers. The values of the score lied between 13 and 65 points. This variable was classified by the tertile boundaries.

*Psychosocial factors* were ascertained with the effort-reward imbalance questionnaire (23-Item version) [29]. The psychosocial situation (ERI) and the personality trait of *overcommitment (OVC)* were recorded with 3 scales (*effort*: 6 Items, *reward*: 11 Items and *OVC*: 6 Items). In accordance with the definition, the ERI ratio score was calculated from the ratio of the cumulative scale for *effort* to the cumulative scale for *reward*, with a correction for the different number of items in the two scales: ∑ Effort/∑Reward*0.5454. An effort-reward imbalance is defined as an ERI ratio score greater than unity. Independently of the scale range, increased *overcommitment* was defined as a value in the upper tertile of the distribution (third tertile: 16.6).

Other workplace-related stresses and resources were recorded with selected dimensions of a standardised short questionnaire for job analysis [30]. These include *qualitative workload*, *control*, *collaboration*, *information* and *employee participation*, *integration* and *variety*. All dimensions consisted of several individual items, with a response scale from 1 to 5.

The dimension *Personal Burnout* of the Copenhagen Burnout Inventory [31] served to record the employees' risk of burnout. This scale ranges from 0 to 100: values above 50 are defined as indicating an increased risk of burnout.

*Musculoskeletal symptoms* (MS) in the shoulder, of the neck and the lower back were recorded with the Nordic Questionnaire [32]. Pain prevalence values were defined by the presence of pain on 8 to 30 days during the previous 12 months *as well as* pain during the 7 days preceding the day of the survey. By combining these two criteria, we hoped to improve the detection of recurrent or persistent pain in these regions of the body.

### Statistical Evaluation

The statistical evaluation excluded persons who did not work in child care (e.g. housekeepers or cooks). Group comparisons of normally distributed data were compared with single factor variance analysis. The Kruskal-Wallis test was used as non-parametric test. For bivariate associations between factors and MS (primary research question), odds ratios were calculated from contingency tables. Logistic regression was used for multivariate analysis. The Hosmer and Lemeshow method was used [33]. This employs a stepwise backwards procedure. Variables with p> 0.1 were successively excluded.

For the second research question, bivariate correlations were calculated with the Spearman correlation coefficient. For multivariate analyses, logistic and linear regressions were calculated with the stepwise backwards procedure. For both research questions, the following variables were included in the regression procedure: *work-related resources*, *everyday situations at work*, *ERI*, *OVC*, *subjective noise exposure*, *physical stress*, *weekly working hours*, *type of institution*, *working area*, *physical activity*, *age*, *BMI* and *gender*.

For the variables *ERI* and *OVC*, the models included tests for interactions.

For scales calculated from at least six items the values of the missing items were replaced by the mean value of the available items. If more than half of the individual values of a scale were missing for a participant, the whole scale value was recorded as being missing.

The statistical calculations were performed with the statistics software SPSS Version 22.

## Results

Two hundred and thirty of the 400 employees contacted returned the questionnaires (response rate: 57%). Seventeen employees who worked less than ten hours a week and 14 persons working as housekeepers or cooks were subsequently excluded. Thus the data from 199 employees were included in the analysis.

Table 1 describes the characteristics of the employees. The participants in the survey were predominantly women (86.4%). The most frequent age group was between 40 and 50 (29.6%). The mean age was 40 years (not included in the Table). More than 90% were German. 40.2% of the participants had a BMI of at least 25 and 45.7% reported regular physical activity. Almost half (48.7%) worked full-time. Only a few (13.1%) worked in management or administration, but the rest exclusively in child care (86.9%). 56.3% of the participants worked in child day care centres; 26.6% worked in school co-operations and a small proportion (10.6%) in facilities for child and adolescent support. The years of employment could not be evaluated as too many values were missing.

**Table 1:** Description of the sample.

Variable	N	Percentage
***Gender***		
Female	172	86.4%
Male	26	13.1%
Missing	1	0.5%
***Age in years***		
18–30	46	23.1%
30–40	51	25.6%
40–50	59	29.6%
50+	41	20.6%
Missing	2	1.0%
***Nationality***		
German	184	92.5%
Other	15	7.5%
Missing	0	0.0%
***BMI***		
**>25**	115	57.8%
≥25	80	40.2%
Missing	4	2.0%
***Physical activity***		
Regular	91	45.7%
None	108	54.3%
Missing	0	0.0%
***Weekly working hours***		
Full-time	97	48.7%
Part-time	102	51.3%
Missing	0	0.0%
***Working area***		
Management/Administration	26	13.1%
Child Care	173	86.9%
Missing	0	0.0%
***Institution***		
Child Day Care Centre	112	56.3%
School Co-operation	53	26.6%
Child and Adolescent Support	21	10.6%
Missing	13	6.5%
**Total**	199	100.0%

The frequency of situations in typical everyday working life are listed by frequency in Table 2. Many (88.7%) of the participants reported that the situation at work was very noisy; this was most frequent in child day care centres (93.8%). The second most common problem was that the participants felt that the groups were too large (77.5%). The problem with screaming children was most often reported by employees in child day care centres (80.4%). More than half the participants (58.1%) reported that they regularly had conflicts with parents. This was least common for employees in child and adolescent support. Conflicts with colleagues were most frequently given by employees in child day care centres (59.8%). Inadequate breaks were given by 50% and conflicts with management are given by 31.7% of participants.

**Table 2:** Descriptions of situations in everyday working life.

Situation	Child Day Care Centres		School Co-operations		Child and Adolescent Support		Total	
	%	N	%	N	%	N	%	N
**It is often too loud where I work.** ^a^	93.8%	105	86.8%	46	66.7%	14	88.7%	165
**Our groups are too large.**	79.4%	85	80.4%	41	60%	12	77.5%	138
**There are children who suddenly start screaming and cannot be influenced.**	80.4%	86	71.7%	38	57.1%	12	75.1%	136
**There are conflicts with parents.**	60.7%	68	60.4%	32	38.1%	8	58.1%	108
**There are conflicts with colleagues.** ^a^	59.8%	67	41.5%	22	28.6%	6	51.1%	95
**Breaks are inadequate.**	42.7%	47	60.4%	32	61.9%	13	50.0%	92
**There are conflicts with management.**	35.8%	39	22.6%	12	33.3%	7	31.7%	58
**Total**	100%	112	100%	53	100%	21	100%	186

^a^: p < 0.01

Table 3 shows the mean values for the different resources and stress scales. For the whole group, the values of the resources were always about 75% of the maximal possible values. The employees of child and adolescent support generally exhibited the highest means. There were statistically significant differences in physical stress and subjective exposure to noise; the employees in child day care exhibited the highest stress levels.

**Table 3:** Resources and stresses in the institutions.

Resource/Stress	Total		Child Day Care Centres	School Co-operations	Child and Adolescent Support	
	**$\bar{x}$**	SD	****$\bar{x}$****	****$\bar{x}$****	**$\bar{x}$**	P
**Control (Scale: 1–5)**	3.7	.84	3.8	3.3	3.8	0.001
**Variety (Scale: 1–5)**	3.8	.76	3.8	3.7	4.3	0.004
**Integration (Scale: 1–5)**	3.7	.75	3.8	3.5	3.8	0.034
**Collaboration (Scale: 1–5)**	3.7	.66	3.7	3.5	4.1	0.003
**Information and Employee Participation (Scale: 1–5)**	3.8	.72	3.8	3.8	3.9	0.907
**Qualitative Workload(Scale: 1–5)**	2.3	.78	2.3	2.1	2.4	0.093
**Physical Stress(Scale: 5–20)**	14.2	2.7	15.2	12.5	13.1	0.001
**Subjective Noise Exposure (Scale: 13–65)**	39	10.3	41	37	32	0.001

**Fig 1:**
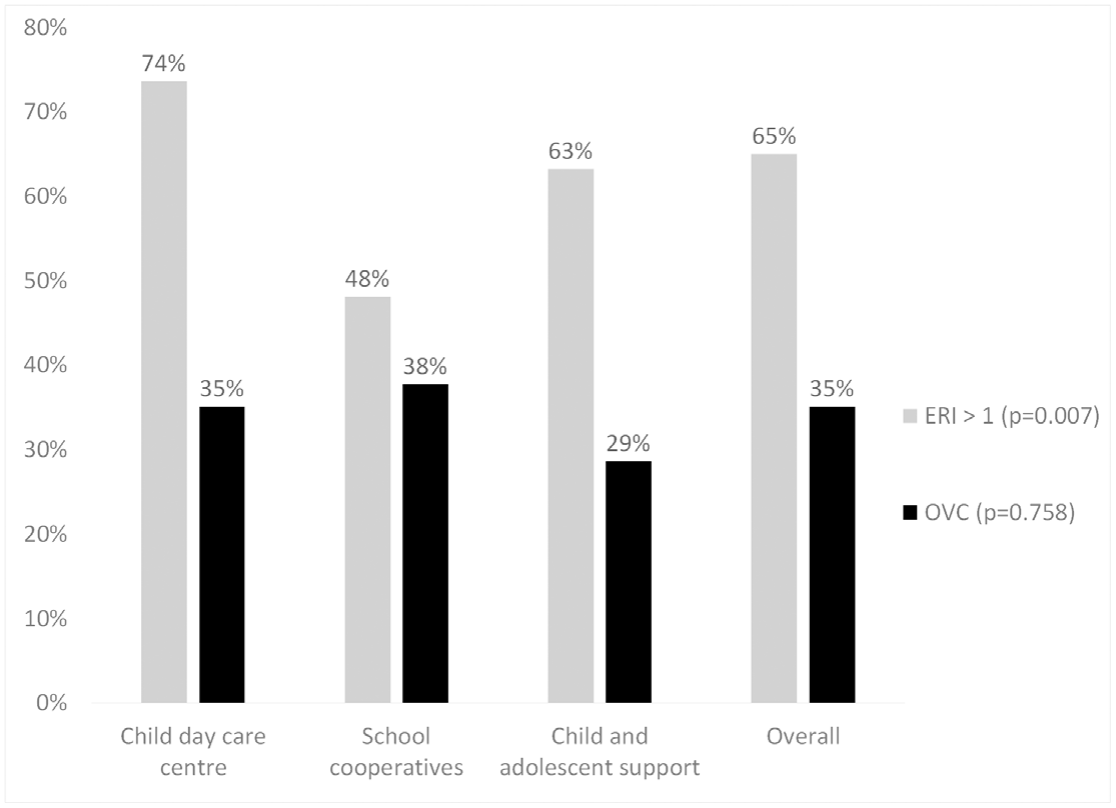
Distribution of the ERI components in the different institutions.

The prevalence of ERI was 65% (mean: 1.17, SD: 0.37) for the whole group. On the other hand, OVC was less prevalent, with 35% (Fig 1). ERI prevalence was greatest for employees in child day care centres (74%). OVC was most frequent in employees of school co-operations (38%).

Persons with increased OVC more often exhibited an ERI ratio score above unity in comparison to persons with low OVC (87% vs. 55%) (data not shown in Fig 1). This difference was statistically significant. According to this, approx. 30% of the overall group exhibited increased OVC and ERI at the same time.

### Risk of Burnout

A risk of burnout (mean: 51.7, SD: 18.2) was observed for most of the sample (56.8%). The risk of burnout was greatest (64.9%) for employees in child care centres (mean: 54.8, SD: 18.3).

Correlations were calculated between the continuous factors and the risk of burnout score. The highest statistically significant correlations were found for *OVC* (r = 0.49), *subjective noise exposure* (r = 0.49) and the *ERI ratio score* (r = 0.45). Weaker correlations were found for *effort score* (r = 0.42), *reward score* (r = -0.38), *information and employee participation* (r = -0.35), *qualitative workload* (r = 0.35) and *physical stress* (r = 0.32).

To fulfil the precondition for the linear regression procedure, the ERI variable had to be converted to logarithms. Table 4 shows the standardised beta coefficients and the significances of the final model. *Subjective noise exposure* (beta: 0.315, p: 0.001), *OVC score* (beta: 0.212, p: 0.006) and *qualitative workload* (beta: 0.145, p: 0.044) showed statistically significant relationship to burnout. The logarithm of the *ERI ratio score* exhibited no relevant influence on the outcome (beta: 0.083, p: 0.304).

**Table 4:** Results of the linear regression for the outcome risk of burnout.

	Beta Coefficient ^a^	p
**Qualitative Workload**	**0.145**	**0.044**
**Subjective Noise Exposure**	**0.315**	**0.001**
**Weekly Working Hours**	0.132	0.057
**Groups too large**	0.125	0.063
**ERI Ratio Log Score**	0.083	0.304
**OVC Score**	**0.212**	**0.006**

^a^: Standardised beta coefficient adjusted for age, gender and institution, R^2^: 0.425

Table 5 presents the results of the multivariate logistic regression. For persons with increased *OVC*, there was a statistically significant increased risk of burnout (OR: 3.4; 95%CI: 1.46–7.75). However an increase in *ERI* *≥* *1* had no relevant effect (OR: 1.2; 95%CI: 0.54–2.70). There was a trend in the risk estimates for *subjective noise exposure*. For persons with subjective noise exposure in the third tertile, there was a statistically significant risk increase (OR: 4.4; 95%CI: 1.55–12.29). No statistically significant interaction effect between *ERI* and *OVC* was found in any regression model. The logistic regression model was also calculated using an ERI variable with the highest tertile as cut-off point. No statistically significant increase was found in the odds ratio.

**Table 5:** Results of the multivariate logistic regression for risk of burnout.

Variable	Specification	OR	95%-CI	p
**Variety**	High	1	.	.
	Little	2.1	0.98–4.32	0.055
**Subjective Noise Exposure**	1.Tertile	1	.	.
	2.Tertile	1.9	0.78–4.83	0.150
	3.Tertile	**4.4**	**1.55–12.29**	**0.005**
**Physical Stress**	1.Tertile	1	.	.
	2.Tertile	1.2	0.37–3.84	0.770
	3.Tertile	2.3	0.97–5.50	0.058
**Weekly Working Hours**	Part time	1	.	.
	Full time	1.9	0.85–4.43	0.118
**ERI**	< 1	1	.	.
	≥ 1	1.2	0.54–2.70	0.649
**OVC**	1./2.Tertile	1	.	.
	3.Tertile	**3.4**	**1.46–7.75**	**0.004**

### Musculoskeletal Symptoms

**Fig 2:**
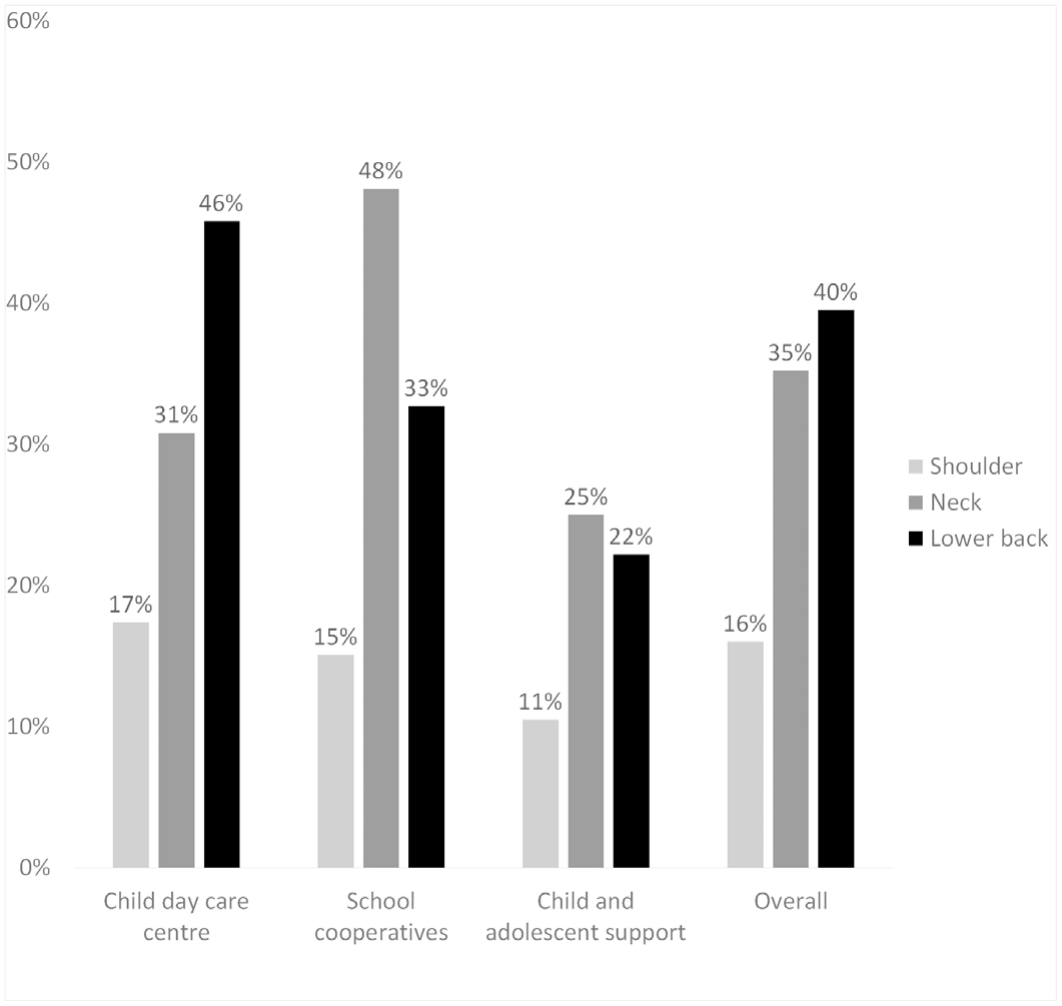
Prevalence of musculoskeletal symptoms.

Fig 2 shows the prevalence for chronic or recurrent MS. Pain in the lower back was most often reported (40%), followed by pain of the neck (35%) and shoulder (16%). The highest prevalence for lower back and shoulder pain were reported for child day care centres (46% or 17%, respectively). The greatest value for neck pain were found for employees of school co-operations (48%).

Table 6 shows the results of the multivariate logistic regression. For the outcome of low back pain, there was a statistically significant increased odds ratio for *OVC* of 2.2 (95%CI: 1.04–4.51). For the outcome of shoulder pain, there was a statistically significant increased odds ratio for *low control* (OR: 3.5; 95%CI: 1.31–9.27). For the neck pain, there were statistically significant increased odds ratios for both *low control* (OR: 4.3; 95%CI: 2.02–9.10) and *risk of burnout* (OR: 2.7; 95%CI: 1.16–6.08).

**Table 6:** Final models of multivariate logistic regression for MS.

	Low Back		Shoulder		Neck		Total MS	
	76 (40%)		32 (16%)		68 (35%)		107 (59%)	
Variable	OR 95% CI	p	OR 95% CI	p	OR 95% CI	p	OR 95% CI	p
Low Control	—		**3.5 (1.31–9.27)**	**0.012**	**4.3 (2.02–9.10)**	**0.001**	**3.8 (1.68–3.37)**	**0.001**
ERI ≥1	1.0 (0.46–2.17)	0.991	0.7 (0.23–1.89)	0.447	0.8 (0.35–1.86)	0.629	0.8 (0.35–1.92)	0.663
OVC Highest Tertile	**2.2 (1.04–4.51)**	**0.038**	1.0 (0.36–2.56)	0.953	0.8 (0.35–1.71)	0.537	1.0 (0.43–2.31)	0.998
Working Area: Management vs. Child Care Area	**—**		**—**		—		**4.5 (1.06–19.28)**	**0.041**
Screaming Children	**—**		—		—		**2.7 (1.01–7.21)**	**0.047**
Risk of Burnout	2.0 (0.96–4.25)	0.063	**—**		**2.7 (1.16–6.08)**	**0.021**	**2.3 (1.01–5.28)**	**0.046**

For the outcome of overall prevalence of MS, there were statistically significant increased odds ratios for *low control* (OR: 3.8, 95%CI: 1.68–3.37), *management* (OR: 4.5, 95%CI: 1.06–19.28), *screaming children* (OR: 2.7, 95%CI: 1.01–7.21), as well as for persons at *risk of burnout* (OR: 2.3, 95%CI: 1.10–5.28).

No statistically significant interaction effects between *ERI* and *OVC* were found in any regression model. All models were also calculated with an ERI variable with the highest tertile as cut-off point. Here too no statistically significant increases were found in the odds ratios.

## Discussion

Factors such as subjective noise, qualitative workload and OVC showed significant relationships to risk of personal burnout in child care workers.

We found significant associations between low control at work and pain in shoulder, neck and total MS. Risk of burnout was associated with neck pain and total MS. For persons with *ERI ratio score > 1* no statistically significant increase in the odds ratios in any region of the body or for risk of burnout were found.

### Effort-Reward Imbalance

Nübling et al. [20] observed a mean ERI ratio score of 0.6 for the social professions. We found a mean of 1.17 for child care workers, with a prevalence of 65% for an ERI ratio score > 1 —a comparatively high value. In an older study with child care workers, Scheuch & Seibt [16] also found a lower mean value of 0.5. On the other hand, some current German studies have also found high prevalence values for ERI, including 64% and 67% for teachers and 87% for managers of child care workers [18, 19]. These high prevalence values for ERI could possibly be the result of the expansion of child care within Germany in the past few years, resulting in excessively large groups of children and short term employment contracts. This can, for example, lead to high exposure to noise and more frequent conflict situations in everyday working life.

### Musculoskeletal Symptoms

In the context of the present study, no association was found between *ERI ratio score >1* and MS in child care workers. The analysis corrected for the confounder *physical stress*. Even a more stringent classification of ERI by splitting into tertiles [34–36] did not lead to any relevant effects in this study. The observed association between *OVC* and low back pain has been demonstrated in the literature. Bernard et al. [37] found relevant effects in vineyard workers and von der Knesebeck et al. [38] in policemen. Moreover, other studies have found statistically significant associations between *OVC* and MS in other body regions [35, 39–41].

The observed effects for *low control* and shoulder pain, neck pain and total MS are consistent with two reviews. These summarised longitudinal studies and also found associations for pain in the back, neck, shoulder and upper extremities [42, 43]. The demand-control model [44] defines *high demands* and *low control* as unfavourable psychosocial factors. The statistically significant associations with *low control* found in the present study are in contrast with the lack of associations with the psychosocial factors of the ERI model. Excluding *low control* from the multivariate model had only a minimal effect on the odds ratios for *ERI* and *OVC*. In the present study, the dimension *control* was not recorded with the original demand-control questionnaire [30]. Nevertheless we found that the proxy variable *control* is evidently associated with MS, as it records a psychosocial factor that is not included in the ERI model. There is already published evidence that the psychosocial factors in the ERI model and those in the demand-control model are complementary [45, 46].

Persons at *risk of burnout* showed higher odds in neck pain and total MS. Burnout lies at the end of the chain of stress and represents a state proceeded by permanent exposure to stress. It is possible that the scale *Personal Burnout* in the present study more specifically identifies the persons exposed to long-term occupational stress factors and who are therefore at increased risk of MS. The association between burnout and MS has been described in longitudinal and cross-sectional studies [47–49]. Armon et al. [47] and Melamed [48] found statistically significant effects in white collar workers and in factory workers respectively in longitudinal studies. Kozak et al. [49] described the statistically significant association between burnout and MS in a cross-sectional study in veterinarians.

An increased odds ratio of 4.5 was found for *managers*. This effect is difficult to interpret, as managers tend to work in offices and only rarely have to sit in awkward postures in chairs intended for children. It may be that managerial work is associated with other psychosocial or organisational stress that is linked to MS, but which was not recorded separately.

### Risk of Burnout

In a summary of burnout research [1], burnout is described as “a well documented phenomenon in the service provision sector”. In the present study we found a considerably high risk of burnout (≥ 50 points: 57%, $\bar{x}$: 52). Reference data for 2013 from the COPSOQ Database give a mean value of 48 for child care workers (data in S1 Supporting Information). Buch & Frieling [8] give the risk of burnout of German child care workers as 30%.

The effects observed in linear regression were confirmed in logistic regression in two of three cases (*OVC* and *subjective noise exposure)*.

The statistically significant association between *OVC* and risk of burnout (OR: 3.4) has been observed in managerial staff [40] and also across the different occupational groups [20]. This association has also been demonstrated in health service employees and in teachers [50, 51]. It seems plausible that an underlying attendance for excessive working is a premise for the development of burnout symptoms.

In the present study, *subjective noise exposure* was not recorded with a validated instrument, but with a questionnaire specially developed for this setting. Nevertheless, the observed association with risk of burnout appears to be reliable; in both regression procedures, *subjective noise exposure* exhibits the greatest effect estimates in the model. The evidence for a trend in the risk estimates in the logistic regression model also supports a robust association with the burnout risk. Child care workers are exposed to a special sort of noise. Studies have shown that loud speech requires more attention and cognitive effort than meaningless noise, e.g. noise from machines [52]. This also applies when the information content of the speech is irrelevant for the listeners. In the present study, 77% of participants reported that the groups were too large and 88% that it was often too loud. We assume that there is massive stress from this type of noise. Other studies in child care workers report mean individual noise levels between 71 and 83 db(A) on one working day, maximum levels reaching levels above 100 db(A) [12–14]. Although our study does not assess objective noise levels, the correlation between subjective noise perception und measured individual noise levels is known [53].

For child care workers and teachers an association between subjective or objective noise and burnout has been observed elsewhere [12, 54]. Sjodin et al. [12] conclude that subjective noise exposure does not contribute do the development of burnout. Furthermore this association shows that workers with an elevated risk of burnout tend to develop an increased sensitivity to noise. Therefore they are more vulnerable to noise. However, known adverse health effects due to noise exposure are coronary heart disease, hypertension, stress and sleep disturbance [55–58]. In the context of day care centres high noise levels may cause auditory fatigue [59]. Furthermore, from the children’s view continuous noise levels reduce intelligibility and verbal acquisition amongst children in classrooms [60]. This report assumes the results of an occupational health risk assessment. However, children’s health is at least just as important in this setting, but not the focus of this paper.

The dimension of *qualitative workload* employs two items to record the concentration required and the complexity of the work. The observed—albeit low—association with risk of burnout shows the challenges of having to concentrate. Potentially this association can also be explained by an inverse causality: child care workers with increased risk of burnout experience a major challenge performing work tasks that require high levels of concentration, e.g. listening to informative noise.

In general, strikingly high prevalence values were found in this study, particularly ERI, OVC, risk of burnout, MS and situations in everyday working life. The responses may have been influenced by current changes in the occupational situation of child care workers in Germany. The results may have been biased by long-term dissatisfaction at work, the wish to change the underlying conditions, as well as social desirability. It is possible that the results would have been similar for another sample from another location in Germany at the same point in time.

### Limitations

The response rate was 57%, which was a relatively good value for a group of employees. However, a non-responder analysis was not performed, so it is not possible to conclude whether the missing group of persons was distinct from the sample in any way.

Due to the cross-sectional design, it is not possible to establish causality. It is theoretically possible that the direction of the causality is the opposite of what we have assumed. A longitudinal study has demonstrated that MS is a predictor of effort-reward imbalance [61]. A bias from common-method variance is likely, e.g. from social desirability [62]. Concededly no objective noise measures were performed in this study. Subjective noise exposure, however, was detected as a relevant factor.

Work-related psychosocial factors are a part of working life. We did not control for the effect of psychosocial factors on private life, e.g. stress in the family. We also failed to consider other factors in the overall psychosocial situation of the employees that complement the factors in the ERI model. For example, these include the entire components of the job demand-control model [44]. Moreover, the odds ratios in this study are overestimated. As the prevalence values of the outcome variables are high, the risk estimates must be interpreted with caution.

### Conclusion

The study results provide risk assessments that permits the inference of specific objectives and interventions for workers in different institutions. Workers with a high risk of burnout appear as vulnerable workers. At the level of the individual place of work, objectives should concentrate on noise and the resource control, as both of these factors may influence the well-being and both can be changed. Historically child care workers were not considered to be have a noisy workplace. However, this study provides new results implicating that occupational safety and health protection should be modified accordingly. Suitable measures for reducing noise should be introduced to the workplaces and then evaluated. In addition, the possibilities should be determined of enhancing control in organisation at work. The existing situation should be analysed and possibly reorganised.

## Supporting Information

S1 Supporting information Statement Personal Burnout COPSOQ Database. (PDF)

## Abbreviations

ERI: effort reward imbalance
MS: musculoskeletal symptoms
OR: odds ratio
OVC: overcommitment
SD: standard deviation
95%-CI: 95% confidence interval
