# Supplementary material for: Musculoskeletal Symptoms and Risk of Burnout in Child Care Workers — A Cross-Sectional Study
Source: PLoS One. 2015 Oct 21;10(10):e0140980. doi: 10.1371/journal.pone.0140980 (PMC4619302; doi:10.1371/journal.pone.0140980)
Supplement: S1 Supporting information — (PDF) [file pone.0140980.s001.pdf]

Freiburg, 09.07.2015

Dear Peter Koch,

I herewith confirm the following:
Mr. Peter Koch has the permission to present unpublished data about personal burnout of child care workers of the German COPSOQ database.
Mean value for CBI, personal burnout is 48 point on a 0-100 scale, date:
January 2013.

best regards
Matthias Nübling

Dr. Mattias Nübling
Head of COPSOQ studies Germany
FFAW GmbH: Freiburg research centre for occupational sciences Bertoldstr. 63
D- 79098 Freiburg
T: 0049 (0)761 894421
F: 0049 (0)761 83432
M: 0046 (0)160 7868616
nuebling@ffaw.de
www.ffaw.de www.copsoq.de
